# Sleep disorders during pregnancy: an underestimated risk factor for gestational diabetes mellitus

**DOI:** 10.1007/s12020-023-03537-x

**Published:** 2023-09-23

**Authors:** Danai Eleftheriou, Kleoniki I. Athanasiadou, Emmanouil Sifnaios, Emmanouil Vagiakis, Paraskevi Katsaounou, Theodora Psaltopoulou, Stavroula A. Paschou, Georgia Trakada

**Affiliations:** 1 Respiratory Medicine Unit, Department of Clinical Therapeutics, Alexandra Hospital, School of Medicine, National and Kapodistrian University of Athens, Athens, Greece; 2 Endocrine Unit and Diabetes Centre, Department of Clinical Therapeutics, Alexandra Hospital, School of Medicine, National and Kapodistrian University of Athens, Athens, Greece; 3 Allergy & Clinical Immunology, Private Clinic, Kalamata, Greece; 4 Respiratory Department, First ICU Evangelismos Hospital Athens, National and Kapodistrian University of Athens, Athens, Greece

**Keywords:** GDM, Gestational diabetes mellitus, Insomnia, Narcolepsy, Obstructive sleep apnea, RLS

## Abstract

Sleep disorders are highly prevalent during pregnancy and significantly affect women’s health and quality of life. Gestational diabetes mellitus (GDM) is one of the most common metabolic complications during pregnancy and constitutes a significant risk factor for both mother and fetus in the short and the long term. While the association between sleep disorders and type 2 diabetes mellitus (T2DM) is indisputable, it is not clear whether there is a link between sleep disorders and GDM. The aim of this article was to investigate the association between sleep disorders and GDM and whether the treatment of sleep disorders may prevent GDM development. Insomnia, obstructive sleep apnea (OSA), restless legs syndrome (RLS), and narcolepsy were the most common sleep disorders identified during pregnancy and were related to poor sleep quality and short or prolonged sleep duration. They were all associated with an increased risk of GDM. The ideal sleep duration for pregnant women was determined at 8–9 h daily. In conclusion, sleep disorders constitute a risk factor for GDM. It is imperative that prospective studies be conducted to evaluate the effect of the early management of sleep disorders on GDM manifestation and control. Healthcare providers should highlight the importance of sufficient sleep to reinforce pregnancy outcomes.

## Introduction

Sleep is an essential function that preserves life and supports mental and physical health. The absence of sleep is incompatible with life. Sleep disorders are common health conditions that usually affect the quality, timing, and duration of sleep resulting in dysfunction, fatigue, and sleepiness during the day [1]. Sleep disorders are undermining the quality of life and have been associated with the development of various medical conditions, such as type 2 diabetes mellitus (T2DM), cardiovascular disease (CVD), non-alcoholic liver disease (NAFLD), and dyslipidemia [2]. However, their association with gestational diabetes mellitus (GDM) is not yet established. GDM is the most common metabolic complication of pregnancy in which the advanced maternal age (≥35 years), maternal obesity (body mass index, BMI ≥ 30 kg/m^2^), personal history of GDM in a previous pregnancy, or the family history of diabetes are the most prominent risk factors [3]. GDM screening is performed in every pregnant woman between 24–28 gestational weeks with an Oral Glucose Tolerance Test (OGTT). The main diagnostic methods that are currently used are (1) the 75-g OGTT proposed by the International Association of Diabetes and Pregnancy Study Group (IADPSG), or (2) the older two-step approach of Carpenter and Coustan that includes an initial 50-g screening test, followed by 100-g OGTT for the positive subjects [4, 5]. GDM is related to multiple gestational, perinatal, and future complications for the mother and the offspring, and these pregnancies are classified as high-risk. The present study aims to investigate the association between sleep disorders and GDM.

### Sleep disorders during pregnancy

#### Insomnia

Insomnia during pregnancy has a high incidence, reaching up to 61%, and deteriorates with the advance of gestational age. Reported insomnia has been associated with GDM, depression, pre-eclampsia, fetal growth restriction, preterm delivery, prolonged labor, cesarean section, and undiagnosed underlying psychiatric disease [6]. In treating insomnia, efforts should focus on educating pregnant women on sleep hygiene, exercise, and behavioral therapy, while the aid of a psychologist-psychotherapist may be essential. Few pharmaceutical substances, such as the antihistamine diphenhydramine, could be used in pregnancy [7].

#### Sleep-related breathing disorders

The most common sleep-related breathing disorder during pregnancy is obstructive sleep apnea (OSA) or obstructive sleep apnea-hypopnea syndrome (OSAHS). Various questionnaires, such as Berlin Questionnaire (BQ), Epworth Sleepiness Scale (ESS), STOP and STOP-BANG, are used as screening tools; however, none of these has a high sensitivity [8]. Fatigue and daytime sleepiness are the most common clinical features while snoring, awakenings with dyspnea, nocturnal polyuria, and apneas observed by partners are also present. OSA in pregnancy has been associated with various complications, such as GDM, hypertension, pre-eclampsia, premature labor, cardiomyopathy, congestive heart failure, low birth weight, reduced Apgar score, postpartum neonatal intensive care unit (NICU) hospitalization and prolonged hospitalization. However, a common confounder is the coexistence of obesity. In addition to OSA, even simple snoring, the incidence of which increases in pregnancy, has been associated with GDM, hypertension, and small-for-gestational-age (SGA) fetuses [9].

#### Restless legs syndrome (RLS)

Restless legs syndrome (RLS) is characterized by an unpleasant urge to move one’s legs and affects the onset and maintenance of sleep. Hemoglobin values below 11 g/dl, family history of RLS, and age ≥35 years are some known risk factors. Moreover, RLS has been associated with a higher risk of GDM, poor sleep quality, daytime dysfunction, drowsiness, pre-eclampsia, preterm delivery, low birth weight, and depression. The treatment of choice is non-pharmaceutical, including exercise, massage, and the use of pressure devices. Iron administration to restore the ferritin value to at least 75 mcg/l is considered essential [10].

#### Narcolepsy and other sleep disorders

Narcolepsy is characterized by sudden involuntary sleep episodes. Women with an increased BMI before and those with excessive weight gain during pregnancy face a higher risk of developing narcolepsy type I or narcolepsy with cataplexy, a sudden muscle weakness triggered by laughing, crying or terror. There are no official recommendations for the treatment of narcolepsy with first onset during pregnancy since all the available medications are not authorized for such use [11]. However, a recent consensus by sleep experts concluded that the risk of teratogenic effects from narcolepsy medications is extremely low [12].

### Sleep duration and quality and risk of GDM

Sleep evaluation is performed with either subjective or objective measures. The subjective assessment includes structured or official questionnaires, while objective assessment is implemented with actigraphy or a sleep study, also known as polysomnography (PSG).

#### Sleep evaluation using questionnaires

Qiu et al. evaluated the association between sleep duration and snoring during pregnancy with the risk of GDM in 1290 pregnant women [13]. After adjustment for maternal age and ethnicity, women who reported a sleep duration of ≤4 h per night at the beginning of pregnancy had a 5.56 times greater risk of developing GDM than women with a sleep duration of 9 h. It also appeared that even an extra hour of sleep reduced the risk for GDM by 15%. Moreover, women who reported sleep duration ≥10 h had higher plasma glucose concentrations, while snoring was related to a 1.86-fold increased risk of GDM. Rawal et al. published a study of 2581 pregnant women evaluating the association of sleep duration in the 1st and 2nd trimesters with GDM development using structured questionnaires [14]. Sleep duration decreased gradually in ≤7 h from the 1st to the 2nd trimester, along with a reduction in daytime sleep intervals. There was no association of sleep duration in the 1st trimester with the onset of GDM. It was observed that non-obese pregnant women who slept more or less than 8–9 h in the 2nd trimester had twice the risk of developing GDM, indicating a U-shaped association between GDM and sleep duration. Followingly, Wang et al. studied sleep duration and quality in 12,506 Chinese women, showing that moderate and poor sleep quality during pregnancy was associated with a higher risk of GDM development (OR 1.62 and 1.77, respectively) [15]. According to two recent studies, GDM prevalence was prominent in women with poor sleep quality and nocturnal sleep duration <6 or 7 h (OR 1.96, 1.32, respectively) [16, 17].

Zhong et al. evaluated the association between poor sleep quality and duration in early pregnancy with GDM in 4066 pregnant women [18]. They filled out a structured questionnaire before the 16th gestational week to assess the duration and quality of morning and nocturnal sleep, with a repeat of the questionnaire at 24–28 weeks, when the OGTT was also performed. Poor sleep quality in early pregnancy combined with long sleep duration (≥8.5 h) was associated with an increased risk of GDM (OR 2.27). Another study, including 48,787 pregnant women, concluded that sleep duration less than 5 and more than 10 h was associated with a higher risk of GDM (OR 1.30 and 1.21, respectively) [19]. Wang et al. studied 1300 women and showed that sleep duration <7 or >10 h and poor sleep quality were associated with a higher risk of GDM (OR 4.28, 4.42 and 1.75, respectively). The combination of poor sleep quality and duration of <7 h was related to a greater risk of GDM (OR 12.71) [20]. Peivandi et al. concluded that women with poor sleep quality had significantly higher BMI levels and a higher risk of developing GDM (OR 2.99) [21]. Furthermore, according to another study, no difference was detected in GDM prevalence in women with poor sleep quality, while short sleep duration was associated with an increased risk for GDM [22]. Song et al. concluded that a positive BQ index increased the risk of GDM development from 27.5% to 66.7%, indicating an interaction of OSA with GDM [23]. Conclusively, sleep duration and risk of GDM present a U-shaped association, indicating that the optimal sleep duration during pregnancy is 8–9 h. The various findings are summarized at Table 1.

**Table 1:** Association of sleep disorders with the risk of GDM using questionnaires

Study ID	Country	Population	Gestational age	BMI	Age (y)	Sleep assessment method	GDM diagnostic criteria	Findings
Qiu et al., 2010 [13]	USA	1290	<20 weeks	All	≥18	Structured questionnaire	100-g OGTT (Carpenter-Coustan)	Sleep duration ≤4 h was related with a higher risk of GDM (RR 4.18). Snoring was associated with increased risk for GDM (aRR 1.56)-after adjustment for BMI.
Hayase et al., 2014 [43]	Japan	35	2nd–3rd trimester	18.4–41.7	27–45	PSQI, PSS questionnaires	Derived from registry	GDM and pregnancy advance were associated with worse sleep quality.
Gonzales et al., 2015 [44]	Spain	130	24–35 weeks	25.2 ± 3.44 (GDM group)/28.1 ± 3.92 (control)	33.4 ± 5.8	PSQI, ESS questionnaires	100-g OGTT (NDDG)	GDM was associated with worse sleep quality and daytime dysfunction, as well as higher ESS index.
Rawal et al., 2017 [14]	USA	2581	1st–2nd trimester	17.8–48.8	All	Structured questionnaire	100-g OGTT (Carpenter-Coustan) or according to registry	Sleep duration more or less than 8–9 h in the 2nd trimester was associated with higher risk of GDM. The ideal sleep duration for pregnant women is 8–9 h.
Wang et al., 2017 [15]	China	12,506	24–28 weeks	All	All	Structured questionnaire	75-g OGTT (IADPSG)	Sleep duration <7 and ≥9 h was associated with greater risk of GDM (OR 1.36 and 1.21 respectively); U-shaped association. Moderate and poor sleep quality were related with greater risk of GDM (OR 1.62 and 1.77 respectively).
Cai et al., 2017 [16]	Singapore	686	26–28 weeks	All	≥18	PSQI questionnaire	75-g OGTT (WHO 1999)	Sleep duration <6 h and poor sleep quality were associated with greater risk of GDM (OR 1.96 and 1.75 respectively).
Zhong et al., 2018 [18]	China	4066	<16 weeks (repeat: 24–28 weeks)	All	All	Structured questionnaire	75-g OGTT (IADPSG)	Poor sleep quality and sleep duration ≥8.5 h were associated with higher risk of GDM (OR 2.27).
Myoga et al., 2019 [19]	Japan	48,787	2nd–3rd trimester	All	All	Structured questionnaire	50-g OGTT or random plasma glucose	Sleep duration <5 h was associated with higher risk of GDM (OR 1.30).
Wang et al., 2020 [20]	China	500	1–13 weeks	17–23	25–31	Adjusted PSQI questionnaire	75-g OGTT (IADPSG)	Duration of nocturnal sleep <7 h and ≥9 h was associated with daytime sleepiness and greater risk of GDM; U-shaped association.
Du et al., 2020 [17]	China	3692	<14 weeks	All	All	PSQI questionnaire	75-g OGTT (IADPSG)	Sleep duration <7 h was associated with higher incidence of GDM (OR 1.32).
Wang et al., 2021 [20]	China	1300	<13 weeks	All	All	PSQI questionnaire	75-g OGTT (IADPSG)	Sleep duration <7 or >10 h and poor sleep quality were associated with higher risk of GDM (OR 4.28, 4.42, and 1.75 respectively). The combination of poor sleep quality and duration of <7 h was related with greater risk of GDM (OR 12.71).
Peivandi et al., 2021 [21]	Iran	240	20–24 weeks	<30	All	PSQI questionnaire	75-g OGTT (IADPSG)	Sleep duration <7 h or >9 h was associated with greater risk of GDM (OR 2.99).
Nicoli et al., 2022 [22]	Italy	386	2nd trimester	All	All	PSQI questionnaire	75-g OGTT (IADPSG)	Short sleep duration was associated with increased risk for GDM (aOR 1.55).
Chen et al., 2022 [45]	China	4550	1st trimester	All	All	PSQI questionnaire	75-g OGTT (IADPSG)	Poor sleep quality was associated with a slight increase in the risk of GDM (OR 1.18).
Song et al., 2022 [23]	China	355	1st trimester	All	All	PSQI, BQ questionnaires	75-g OGTT (IADPSG)	The high risk of OSA before pregnancy may increase the risk for GDM during pregnancy.

*aOR* adjusted odds ratio, *aRR* adjusted risk ratio, *BQ* Berlin questionnaire, *ESS* Epworth sleepiness scale, *GDM* gestational diabetes mellitus, *IADPSG* International Association of Diabetes and Pregnancy Study Group, *NDDG* National Diabetes Data Group, *NICE* national institute for health and care excellence, *OGTT* oral glucose tolerance test, *OSA* obstructive sleep apnea, *PSQI* Pittsburgh sleep quality Index, *PSS* perceived stress scale

#### Sleep evaluation using actigraphy

Actigraphy is a non-invasive and objective diagnostic method of sleep duration and quality [24]. Twedt et al. showed that shorter sleep duration has been related to worse glycemic control [25]. Nocturnal sleep duration <7 h and later sleep midpoint were associated with statistically significant increased risk for GDM [26]. Finally, in another study short sleep duration was related to impaired glucose tolerance [27]. The findings are summarized at Table 2.

**Table 2:** Association of sleep disorders with the risk of GDM using actigraphy or polysomnography

Study ID	Country	Population	Gestational age	BMI	Age (y)	Questionnaire	Sleep study	OSA diagnostic criteria	GDM diagnostic criteria	Findings
Reutrakul et al., 2013 [28]	USA	45	2nd–3rd trimester	All (except for >25 in the non-pregnant group)	All	None	Type I	ΑΗΙ > 5	100-g OGTT (Carpenter-Coustan)	Pregnant women with GDM have shorter sleep duration (*p* = 0.02), and higher incidence of OSA.
Bisson et al., 2014 [42]	Canada	52	3rd trimester	<35	>18	PSQI, ESS, RLS	Type II	ΑΗΙ > 5	75-g OGTT (Canadian Diabetes Association)	Pregnant women with GDM have higher incidence of OSA (OR 1.90), more daytime sleepiness, and higher incidence of RLS.
Twedt et al., 2015 [25]	USA	37	2nd trimester	All	18–50	None	Actigraphy	None	100-g OGTT (Carpenter-Coustan)	Women with GDM had shorter sleep duration and sleep fragmentation.
Facco et al., 2017 [26]	USA	782	16–22 weeks	All	All	None	Actigraphy	None	100-g OGTT (Carpenter-Coustan) or 75-g OGTT (IADPSG) or 50-g OGTT	Sleep duration <7 h and later sleep midpoint were independently associated with increased risk for GDM (OR 2.24 and 2.58, respectively).
Facco et al., 2017 [46]	USA	3,245	6–15 and 22–31 weeks	All	All	None	Type III	ΑΗΙ > 5	100-g OGTT (Carpenter-Coustan) or 75-g OGTT (IADPSG) or 50-g OGTT	Pregnant women with OSA have higher risk of developing GDM (OR 3.47 at the beginning and 2.79 at the mid-pregnancy, respectively). There is an increase in the incidence of GDM with increasing AHI.
Wanitcharoenkul et al., 2017 [29]	Thailand	82	26–33 weeks	>25	All	BQ, ESS	Type III	ΑΗΙ > 5	75-g OGTT (IADPSG)	Higher fasting glucose values – increased insulin resistance in more severe OSA (with greater desaturations).
Bublitz et al., 2018 [32]	USA	23	24–32 weeks	All	All	None	Type ΙΙΙ (MediByte device)	AHI > 5	100-g OGTT (Carpenter-Coustan)	No association between OSA and insulin resistance.
Chirakalwasan et al., 2018 [30]	USA, Thailand	23	24–34 weeks	>25	All	BQ, ESS	Type ΙΙΙ (Watch- PAT200)	AHI > 5	75-g OGTT (IADPSG)	Improved insulin secretion and insulin sensitivity in the CPAP group. Women with longer CPAP use showed lower rates of preterm delivery, unplanned cesarean section, and hospitalization in NICU.
Redfern et al., 2019 [27]	UK	49	27–30 weeks	30–40	18–40	None	Actigraphy	None	75-g OGTT (NICE)	Shorter sleep duration was associated with impaired glucose tolerance at 2 h during the OGTT.
Balserak et al., 2020 [33]	USA	92	24–36 weeks	All	18–42	PSQI, ESS, SASS – MVAP index, habitual snoring	Type I	ΑΗΙ > 5	100-g OGTT (Carpenter-Coustan) or 50-g OGTT	Incidence of OSA, frequency of snoring, SASS, and duration of REM sleep were greater in GDM group. PLMI was more frequent in the non-GDM group. Pregnant women with OSA had higher risk of developing GDM (OR 4.71).
Newbold et al., 2020 [34]	Canada	65	24–34 weeks	33 ± 7	35 ± 5	PSQI	Type II	ΑΗΙ > 10	75-g OGTT (Canadian Diabetes Association)	Greater severity of sleep-related breathing disorders was associated with higher nocturnal and morning glucose levels in women with GDM. (66% of women with GDM had AHI > 10).
Reutrakul et al., 2021 [47]	USA, Thailand	81	24–34 weeks	≥25	All	None	Type ΙΙΙ (Watch-PAT200)	AHI > 5	75-g OGTT (IADPSG)	No difference in the metabolomic profile of women with and without OSAHS.
Alonso-Fernandez et al., 2022 [31]	Spain	177	3rd trimester	All	All	ESS	Type I	ΑΗΙ > 5	100-g OGTT (Carpenter-Coustan)	No association of OSA with GDM.
Sanapo et al., 2022 [35]	USA	192	<20 weeks	≥27	>18	None	Type ΙΙΙ (Nox T3 device)	REI > 5	Derived from registry	Pregnant women with OSA have increased insulin resistance.
Serednytskyy et al., 2022 [36]	Spain	51	3rd trimester	All	33–39	ESS	Type I	AHI > 5	100-g OGTT (Carpenter-Coustan)	No difference in daytime sleepiness, snoring, nocturia, morning headache, and sleep duration in women with and without OSA.

*AHI* apnea-hypopnea index, *BQ* Berlin questionnaire, *CPAP* continuous positive airway pressure, *ESS* Epworth sleepiness scale, *GDM* gestational diabetes mellitus, *IADPSG* International Association of Diabetes and Pregnancy Study Group, *MVAP* multivariable apnea prediction, *NICU* neonatal intensive care unit, *OGTT* oral glucose tolerance test, *OSA* obstructive sleep apnea, *OSAHS* obstructive sleep apnea-hypopnea syndrome, *PLMI* periodic limb movement index, *PSG* polysomnography, *PSQI* Pittsburgh sleep quality index, *RLS* restless legs syndrome, *SASS* sleep apnea symptom score

#### Sleep evaluation using polysomnography

Polysomnography (PSG) is the diagnostic gold standard for the majority of sleep disorders. Reutrakul et al. investigated the relationship between OSA and GDM using PSG [28]. GDM was related to shorter sleep duration, higher microarousal index, 4-fold ΑΗΙ, and increased risk for OSA (OR 6.6) compared to women without GDM. OSA was correlated with increased insulin resistance, pancreatic beta-cell dysfunction, BMI, and FPG values in obese pregnant women with GDM [29]. Chirakalwasan et al. measured the effect of a CPAP device in obese women with GDM and OSA [30]. Insulin sensitivity and secretion were improved in the CPAP subgroup. Alonso–Fernandez et al. evaluated 177 pregnant women with and without GDM in the 3rd trimester of pregnancy. Although women with GDM had greater BMI and increased insulin resistance, OSA prevalence did not differ between the two groups [31].

Bublitz et al. focused on the role of inflammation in OSA and GDM. They conducted a sleep study in 23 women with GDM and detected increased levels of interleukins 6, 8, and TNF-a. The researchers suggested that there is an elevated inflammatory profile in this population [32]. Balserak et al. concluded that OSA prevalence, snoring frequency, and REM sleep duration, were significantly higher in women with GDM [33]. In another study in women with GDM, the elevated ΑΗΙ was related to increased nocturnal glucose levels [34]. Sanapo et al. proved that women with OSA presented increased fasting glucose levels, C-peptide, and insulin resistance [35]. Serednytskyy et al. evaluated the coexistence of OSA and GDM and supported that they were related to increased sympathetic activity and IL-1β levels [36]. Conclusively, GDM was more common in women with OSA, while there is also a higher incidence of OSA in pregnant women with GDM. The findings are summarized at Table 2.

### Other sleep disorders and GDM

The association of narcolepsy, circadian rhythm disorders, and RLS with the risk of GDM was evaluated below (Table 3).

**Table 3:** Association of narcolepsy, circadian rhythm disorders, and RLS with the risk of GDM

Study ID	Country	Population	Sleep disorder	GDM diagnostic criteria	Findings
Maurovich–Horvat et al., 2013 [37]	16 sleep centers from 12 European countries	249	Narcolepsy	Questionnaire	Higher incidence of GDM in women diagnosed with narcolepsy type I before or during pregnancy compared to women diagnosed postpartum.
Bisson et al., 2014 [42]	Canada	52	RLS	75-g OGTT (Canadian Diabetes Association)	Higher incidence of RLS in pregnant women with GDM.
Calvo–Ferrandiz et al., 2017 [38]	Spain	100	Narcolepsy	Questionnaire	Higher incidence of GDM in women with narcolepsy with cataplexy.
Weschenfelder et al., 2021 [40]	Germany	234	Circadian rhythm disorders	75-g OGTT (IADPSG)	Higher need for long-acting insulin therapy in women with circadian rhythm disturbances and GDM.
Wilson et al., 2022 [39]	USA	7742	Narcolepsy	Derived from registry	No association between narcolepsy and GDM.
Mubeen et al., 2022 [41]	Pakistan	478	RLS	Derived from registry	Greater risk of GDM in pregnant women with RLS.

*GDM* gestational diabetes mellitus, *IADPSG* International Association of Diabetes and Pregnancy Study Group, *OGTT* oral glucose tolerance test, *RLS* restless legs syndrome

#### Narcolepsy

The incidence of GDM was higher in women diagnosed with narcolepsy type I before or during pregnancy compared to women diagnosed postpartum [37]. Besides, Calvo–Ferrandiz et al. concluded that GDM was more frequent in women with narcolepsy with cataplexy [38]. In a nationwide study performed in the USA, 7742 women with narcolepsy were detected (88% were diagnosed with narcolepsy type II or narcolepsy without cataplexy). However, no association between narcolepsy and GDM was observed [39].

#### Circadian rhythm disorders

Weschenfelder et al. evaluated 235 women with a history of GDM and concluded that pregnant women raising children and having unfavorable work conditions had a higher need for long-acting insulin therapy [40].

#### Restless legs syndrome (RLS)

A recent study revealed a higher frequency of RLS in women with GDM [41]. Bisson et al. compared 26 pregnant women with GDM with 26 controls, concluding that there was a higher incidence of RLS in the GDM subgroup (46% vs 19%) [42].

## Conclusions

In conclusion, the available evidence indicates an association between sleep disorders during pregnancy and GDM. The interaction mechanisms between sleep disorders and GDM are directed towards the presence of systemic inflammation and stimulation of the sympathetic nervous system. It is crucial to design prospective studies using both subjective and objective sleep assessment methods to recognize sleep disorders during the first trimester of pregnancy. Therefore, early medical intervention will be provided, and GDM prevalence and pregnancy complications will be diminished, ensuring an improvement in public health and the quality of life of pregnant women. Healthcare providers should highlight the importance of sufficient sleep to reinforce pregnancy outcomes. The literature reports of other sleep disorders in pregnancy are scarce, making this field an area of particular interest for future studies. The underlying mechanisms correlating sleep disorders with GDM development should be investigated with further research.
